# MiR-362-5p as a novel prognostic predictor of cytogenetically normal acute myeloid leukemia

**DOI:** 10.1186/s12967-018-1445-3

**Published:** 2018-03-14

**Authors:** Qiu-Ling Ma, Jing-Han Wang, Min Yang, Huan-ping Wang, Jie Jin

**Affiliations:** 1 Department of Hematology, The First Affiliated Hospital, Zhejiang University of Medicine, No. 79 Qingchun Road, 310003 Hangzhou, Zhejiang People’s Republic of China; 2 Key Laboratory of Hematopoietic Malignancies, Hangzhou, Zhejiang Province People’s Republic of China; 3 Department of Hematology, The Second Affiliated Hospital of Henan University of Traditional Chinese Medicine, Zhengzhou, Henan People’s Republic of China

**Keywords:** miR-362-5p, Acute myeloid leukemia, Gene expression, Prognosis

## Abstract

**Background:**

MicroRNAs are of special interest in cancer research and hold significant promise as diagnostic and prognostic biomarkers for malignant disease. MiR-362-5p have been found to exert both oncogenic and tumor suppressive effects depending highly on the cellular context. The aim of this study was to determine whether the expression of miR-362-5p can be served as a prognostic factor for patients with cytogentically normal acute myeloid leukemia (CN-AML).

**Methods:**

We enrolled 224 patients with CN-AML and measured the expression of miR-362-5p by quantitative real time PCR analysis. We classified patients into high and low expression based on the median value. The Cox regression analyses were carried out to assess the prognostic significance of miR-362-5p expression in the context of the well-established predictors. Additionally, microRNA expression profiling were conducted to identify the biological insights between high and low group.

**Results:**

High expressers had older age. High expressers obtained shorter overall survival in the univariate analysis. The independent prognostic value of miR-362-5p remained in the context of the well-established clinical and cytogenetic predictors. Moreover, the prognostic value of miR-362-5p was also validated in an independent cohort of CN-AML. Notably, numerous oncomiRs were also high expressed in high miR-362-5p group.

**Conclusion:**

High miR-362-5p expression was associated with poorer overall survival implicating the oncogenic function in AML development.

## Background

Acute myeloid leukemia (AML) is a group of heterogeneous hematologic malignancy with great variability in molecular signatures, genetic phenotype, prognostic behaviors and treatment outcome [1–4]. Although chromosomal abnormalities have been validated as an effective tool for the classification and risk stratification, chromosomal lesions are identified in approximately 50% of all AML when assessed using conventional banding analysis. In contrast, about 50% of all AML cases are cytogenetically normal (CN). In order to refine classification for CN-AML patients, molecular diagnosis, such as NPM1, FLT3-ITD, CEBPA mutational analysis, is crucial [5, 6]. Beside the genes mutations, oncogenes expression also obtained the prognostic significance in CN-AML [4]. For example, low expression of the brain and acute leukemia cytoplasmic (BAALC) and ETS-related gene (ERG) genes have been associated with better outcome in CN-AML patients [7, 8]. Recently, we found that high expression of HIP1 and IDH1 were associated with poor survival in AML [9]. In addition to genetic alterations in protein coding genes, aberrant expression of noncoding RNAs might play an important role in leukemia initiation and outcome prediction [10].

MicroRNA (miRNA) is the first class of noncoding RNAs and constitutes about 19–22 nucleotides that is involved in post-transcriptional regulation of genes and play important roles in biological functions such as cell differentiation, proliferation, DNA repair, cell adhesion, motility, and apoptosis [11, 12]. The aberrant expression of miRNAs is identified in various disease and cancer [13]. Numerous studies revealed that some miRNA expression significantly influence apoptosis, block hematopoietic differentiation, induce leukemia transformation and drug resistance by controlling gene expression which associated with diagnosis, prognosis, and response to treatment in leukemia [14].

Notable examples of these miRNAs are given by miR-155, miR-29, miR-150 and miR-196b [15]. Recently, a few studies demonstrated miR-362-5p could act as tumor suppressors by targeting phosphatidylinositol 3-kinase-C2beta and inhibiting proliferation and migration of neuroblastoma cells [16]. On the other hand, miR-362-5p could play an oncogenic role by down-regulating tumor suppressor CYLD in gastric cancer and hepatocellular carcinoma [17, 18]. With respect to hematological malignances, miR-326-5p can promote the malignancy of chronic myeloid leukemia via down-regulation of GADD45alpha [19]. However, the role of miR-362-5p in acute myeloid leukemia remains unclear. Here, we investigate the prognostic value of miR-326-5p in CN-AML patients.

## Methods

### Patients

In this study, we selected 224 patients with de novo CN-AML in the center of Zhejiang Institute of Hematology (ZIH), Hangzhou, China from 2008 to 2015. All patients were well-informed about the study and provided written informed consent to participate in the study. The study was approved by the Institutional Review boards of our hospital. The research was conducted in accordance with the Helsinki Declaration. Patients with CN-AML were included in this study. Bone marrow (BM) samples were obtained at the time of diagnosis. Mononuclear cells were isolated from the BM samples by Ficoll-Hypaque density-gradient centrifugation and subsequently used for mutational molecular analyses. To be considered cytogenetically normal, at least 20 metaphase cells from diagnostic bone marrow had been evaluated. Patients with CN-AML are treated with standard Anthracycline and Cytarabine. With respect to the consolidation therapy, these patients were treated with a high-dose Cytarabine-based chemotherapy.

### Cytogenetic and gene mutation analysis

Cytogenetic and molecular studies were performed at ZIH. The pretreatment BM samples from all CN-AML patients were studied by R-banding analysis. The definition of a cytogenetic clone and descriptions of karyotypes followed the International System for Human Cytogenetic Nomenclature [20].

DNA and RNA samples obtained from mononuclear cells from BM samples at primary diagnosis were extracted as described previously [9]. Mutation analyses of NPM1, FLT3-ITD, CEBPA, DNMT3A, IDH1 and IDH2 were carried out as described previously [9].

Bone marrow (BM) mononuclear cells (MNCS) were purified by Ficoll density gradient centrifugation and were dissolved by RNAiso plus (Takara, Japan). Total RNA was extracted and purified using miRNeasy Mini Kit (Qiagen, USA) following the manufacturer’s instructions. 1 µg total RNA was used for cDNA by All-in-OneTM miRNA First Strand cDNA Synthesis Kit(Gene Copeia, USA). miRNA q-PCR was conducted using ALL-in-one™ miRNA qPCR kit (Gene Copeia, USA). The assay was carried out on an IQ5 Real Time PCR instrument (Bio-Rad, USA), PCR reactions were performed in a total volume of 20 μl containing of 2 μl sample cDNA, 10 μl of 2 × All-in-One qPCR Mix, 2 μl of All-in-one miRNA qPCR Primer (2uM), 2 μl of Universal Adaport PCR Primer (2uM), and 4 μl of cDNA, RNase/DNase free H_2_O. The reactions were incubated in a 96-well plate at 95 °C for 10 min, followed by 40 cycles of 95 °C for 10 s, 58 °C for 40 s and 72 °C for 15 s. Relative quantification was calculated using 2^−ΔΔCT^ [21] and U6 was used for normalization. The primers used for q-PCR were miR-362-5p (5′-ATCCTTGGAACCTAGGTGTGAGT-3′) and U6 (5′-TTCGTGAAGCGTTCCATATTTT-3′).

### MicroRNA experiments

For miRNA profiling, total RNA was extracted and purified using mirVana™ miRNA Isolation Kit (Ambion, Austin, TX, US) following the manufacturer’s instructions. RNA integrity number (RIN) was assessed by an Agilent Bioanalyzer 2100 (Agilent technologies, Santa Clara, CA, US). miRNA expression was performed using the Agilent Human miRNA Microarray Kit Version 16.0. Total RNA (100 ng) was hybridized per sample and processed according to the manufacturer’s instructions. The arrays were scanned by an Agilent Technology G2565BA scanner. The scanned images were gridded and analyzed with Agilent Feature Extraction Software Version 10.7. Raw data were normalized by quantile algorithm, Gene Spring Software 11.0. Each microRNA signature was represented by the average of its expression value of replicate probes.

### Statistical analysis

The main objective of this study was to evaluate the association between miR-362-5p expression and overall survival in patients with CN-AML. We classified CN-AML patients into high and low groups based on the median value of miR-362-5p expression. Patient characteristics were summarized using descriptive statistics, which included frequency counts, median and range. The relationship between miR-362-5p expression and patient characteristics was estimated by the nonparametric test and Chi square test. OS was measured as time from disease diagnosis to death from any cause, or censoring for patients alive at their last follow-up. Only 4 patients received bone marrow transplantation (BMT) in our patients, and OS of these patients were censored at days for patients with BM transplantation. Kaplan–Meier method used in univariate analysis and Cox proportional hazard regression model in multivariate analysis were used to determine the prognostic value of miR-362-5p expression. All statistical analyses were conducted with R statistic package, version 3.3.1 (http://www.r-project.org). *P *< 0.05 demonstrated statistical difference.

## Results

### Clinical characteristics of CN-AML patients with aberrant expression of miR-362-5p

Clinical characteristics of 224 CN-AML patients with high and low miR-362-5p expression were summarized in Table 1. High miR-362-5p expression was predominant in older patients. CN-AML patients with high miR-362-5p expression had higher complete remission rate compared to those with low expressers (67.9% vs. 56.2%, *P *= 0.098), however, the difference did not reach the statistical significance. There was no statistically significant correlation between miR-362-5p expression and other variables including sex, white blood cell counts (WBC), hemoglobin, platelet counts (PLT), percentage of bone marrow blasts, FAB subtypes and genes of FLT3-ITD, CEBPA, NPM1, DNMT3A, IDH1 and IDH2 mutations (Table 1).

**Table 1:** Clinical characteristics of patients with aberrant expressed miR-362-5p

Variables	Low expression	High expression	*P* value
Number	112	112	
Sex, n (%)	60 (53.6)	71 (63.4)	0.175
Age, years^#^	45.50 [33.00, 59.00]	50.50 [41.25, 62.00]	0.048
WBC, 10^9^/l^#^	18.60 [4.79, 64.65]	28.30 [5.80, 93.55]	0.301
Hemoglobin, /l^#^	79.50 [66.00, 95.00]	88.50 [67.00, 105.25]	0.061
PLT, 10^9^/l^#^	38.50 [22.75, 77.25]	46.50 [25.00, 109.50]	0.141
Blast, %^#^	69.00 [54.00, 79.62]	71.50 [48.38, 82.62]	0.920
FAB subtype, n (%)			0.904
M0	9 (8.0)	11 (9.8)	
M1	12 (10.7)	11 (9.8)	
M2	47 (42.0)	50 (44.6)	
M4	14 (12.5)	9 (8.0)	
M5	28 (25.0)	28 (25.0)	
M6	2 (1.8)	3 (2.7)	
Genes mutation, n (%)			
FLT3-ITD	19 (17.0)	24 (21.4)	0.498
CEBPA^DM&^	14 (12.5)	17 (15.2)	0.699
NPM1	21 (18.8)	33 (29.5)	0.085
DNMT3A	10 (8.9)	12 (10.7)	0.823
IDH1	7 (6.2)	12 (10.7)	0.338
IDH2	11 (9.8)	19 (17.0)	0.169
CR rates	76 (67.9)	63 (56.2)	0.098

*WBC* white blood cell counts, *PLT* platelet counts, *DM&* double allele mutations

^#^Median (interquartile)

### Associations of miR-362-5p expression and overall survival

**Fig. 1:**
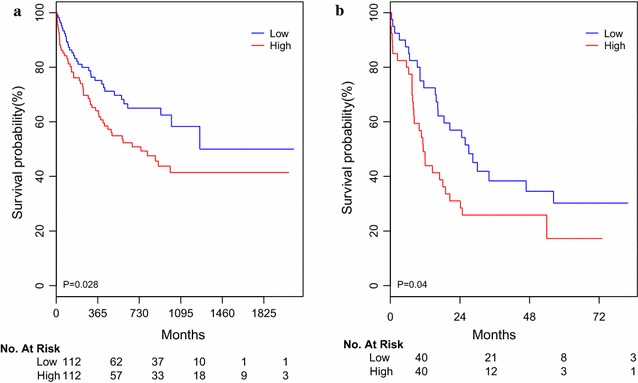
**a** Kaplan–Meier survival analysis of the 224 CN-AML patients. OS curves of cases with high or low level of miR-362-5p expression values based on median value of miR-362-5p expression in CN-AML patients. **b** Kaplan–Meier survival analysis of CN-AML patients as a validation cohort treated according to the TCGA dataset

In this study, the 3-year overall survival (OS) rate of 224 CN-AMLs was 50%. We observed 86 death in this cohort, 44 (51%) death due to no remission, 32 (37%) death from disease relapse, 6 (7%) death from infection to 4 (5%) death because of cerebral hemorrhage during intensive consolidation treatment As shown in Fig. 1a, high miR-362-5p expressers (n = 112) had poorer OS than low expressers (n = 112). Notably, miR-362-5p expression was as an independent prognostic factor in multivariate analysis for OS after adjusting for age, WBC, hemoglobin levels and genes of FLT3-ITD, NPM1, CEBPA, DNMT3A, IDH1 and IDH2 mutations. Additionally, the well-established prognostic factors like age, WBC, and genes of CEBPA and NPM1 mutations were still as the independent prognostic factors in multivariate analysis (Table 2).

**Table 2:** Overall survival analyses for CN-AML patients

Variables	Univariate analysis		Multivariate analysis	
	*P* value	HR (95%)	*P* value	HR (95%)
miR-362-5p	0.028	1.615 (1.048, 2.488)	0.045	1.599 (1.011, 2.528)
WBC	< 0.001	1.005 (1.003, 1.008)	< 0.001	1.006 (1.003, 1.009)
Hemoglobin	0.242	0.995 (0.987, 1.003)	0.297	0.995 (0.987, 1.004)
Age	0.006	1.02 (1.006, 1.035)	0.005	1.022 (1.007, 1.038)
FLT3-ITD	0.654	1.136 (0.65, 1.987)	0.723	1.11 (0.622, 1.982)
CEBPA^DM&^	0.014	0.352 (0.153, 0.809)	0.007	0.312 (0.133, 0.732)
NPM1	0.884	1.038 (0.629, 1.712)	0.039	0.547 (0.309, 0.969)
DNMT3A	0.157	1.58 (0.838, 2.979)	0.356	1.376 (0.699, 2.711)
IDH1	0.043	1.879 (1.019, 3.466)	0.125	1.662 (0.868, 3.183)
IDH2	0.613	0.849 (0.45, 1.601)	0.196	0.644 (0.331, 1.254)

MiR-362-5p: high vs low expression, Age, WBC and hemoglobin are used as continuous variables

*DM&* double allele mutations

In the TCGA dataset, 80 CN-AML patients were classified into high and low group based on the median value of miR-362-5p. We found patients in high miR-362-5p group had poor overall survival than those in low group (Fig. 1b).

### MicroRNA expression profiling

**Fig. 2:**
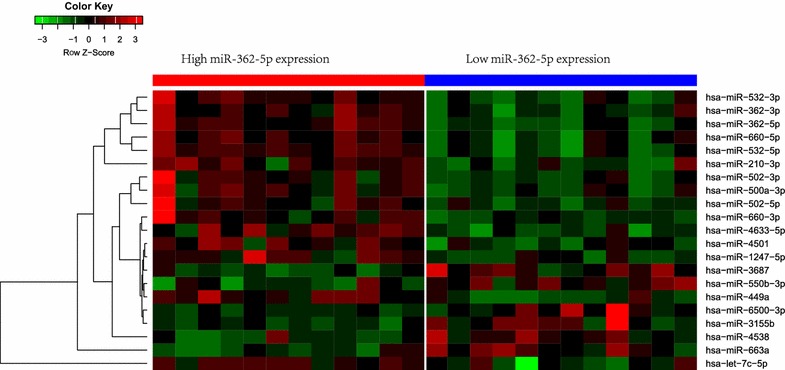
MicroRNA expression patterns in leukemia blasts of AML patients with high miR-362-5p expression compared to those with low miR-362-5p expression

We used 12 samples with high miR-362-5p expression and 12 samples with low expression to assess the differences of microRNA (miR) expression. The most significant changes of miRs in high expressers included up-regulation of miR-532-3p, miR-362-3p, miR-660-5p, miR-532-5p, miR-210-3p, miR-502-3p, miR-500a-3p, miR-502-5p, miR-660-3p, miR-4633-5p, miR-4501, miR-1247-5p, let-7c-5p, miR-449a and down-regulation of miR-663a, miR-4538, miR-3155b, miR-6500-3p, miR-550b-3p, miR-3687 (*P* value < 0.005, Fig. 2).

## Discussion

AML is a highly heterogeneous disease characterized by failure of terminal differentiation from precursor cells into mature blood cells. miRNAs are small, non-coding RNAs that bind target mRNAs leading to their degradation or disruption of cellular proliferation and differentiation in acute myeloid leukemia (AML) [14]. For example, miR-125 and miR-126 control the PI3K-AKT-mTOR pathway, a gatekeeper of leukemic stem cells self-renewal and transient cell-cycle quiescence [22–24]. Additionally, it has been reported that both single miRNA and panel of miRNAs have prognostic significance in patients with AML. Reduced miR-124-1, miR-125, miR-126 expression had longer survival [14]. In contrast, up-regulated miR-320, miR-146, miR-130, miR-223, miR-378 and miR-551b had poor survival in AML [25]. Based on these ground, we believe some miRNAs should act as major players in the development of AMLs and can be used as biomarkers and drug targets.

Recently, miR-362-5p was reported as onco-microRNA in solid tumors [17, 18]. With respect to hematological malignancy, miR-362-5p expression is higher in both CML patient’s samples and cell lines compare to controls [19]. Moreover, the down-regulation of miR-362-5p significantly suppresses chronic myeloid leukemia (CML) cell proliferation, enhances cell apoptosis, induces cell cycle arrest, and decreases migration and invasion in vitro, whereas a miR-362-5p inhibitor reduced tumor volume and tumor growth in vivo in a xenograft model [17]. Furthermore, miR-362-5p inhibitor increases the sensitivity of CML cell lines to the agent Ara-c [17]. On the other hand, 5-Aza-2′-deoxycytidine (DAC, an inhibitor of DNA methylation) significantly decreased methylation of miR-362-5p promoter, leading to increasing expression of miR-362-5p in human hepatocellular carcinoma cells [26]. Taken together, these results indicate that miR-362-5p acts as a novel oncogenic miRNA (oncomiR) that exerts an important effects on solid tumor and leukemia progression. In this study, we examined the expression of miR-362-5p in a large cohort of 224 patients with CN-AML to analyze its prognostic value. In order to test whether it can be used as a reliable prognostic factor, we first classified patients into high and low expressers. We found high expression was predominant in older patients. There were no significant differences between the altered expression of miR-362-5p and the other well-established prognostic factors, such as WBC, hemoglobin and genes of FLT3-ITD, NPM1, CEBPA, DNMT3A, IDH1 and IDH2 mutations. These results indicated that these above factors might not be confounders. When we assumed these well-established factors as potential confounders, we found miR-362-5p expression was still an independent predictor in multivariate models after adjustment of these well-established predictors. Moreover, the prognostic significance was also validated in the TCGA cohort of CN-AMLs. These results indicated that miR-362-5p might act as a reliable and independent predictor in the clinical practice.

It is well known that single miRNA can combine with multiple miRNAs and regulate multiple mRNAs. Therefore, we performed miRNA profiling analysis. As expected, we found two clusters of miRNAs positively and negatively correlated with miR-362-5p expression, respectively. These miRNAs have been proved to be important prognostic markers and novel targets for therapy in cancers. For example, miR-660 expression was used as a good candidate for prognosis prediction in breast cancer [27]. MiR-210 which up-regulated can lead to increased reactive oxygen species as reported by Wei Yang [28]. These clusters of miRNAs are helpful for us to understand the underlying biological insights of miR-362-5p used as a predictors for poor OS.

This study explored the clinical significance of miR-362-5p expression in a large cohort of CN-AML patients in China. However, one potential limitation to our study is the study design were retrospective. In addition, we did not take into account several factors including underlying comorbidities and genes mutations like ASXL1, TET2 in multivariate survival analysis although we excluded the well-established factors as the potential confounders. However, this result was also validated in an independent cohort of CN-AML patients.

## Conclusion

In conclusion, present study demonstrated that cytogenetically normal AML (CN-AML) patients with high expression of miR-362-5p are associated with poorer overall survival implicating the oncogenic function in AML development. This study suggests miR-362-5p can be used as an independent poor predictor in patients with CN-AML.

## Abbreviations

AML: acute myeloid leukemia
CN: cytogenetically normal
BM: bone marrow
MNCS: mononuclear cells
BMT: bone marrow transplantation
NPM1: nucleophosmin-1
FLT3-ITD: Fms-like tyrosine kinase 3 gene internal tandem duplication
IDH1: isocitrate dehydrogenase 1
IDH2: isocitrate dehydrogenase 2
DNMT3A: DNA methyltransferase 3A
CEBPA: CCAAT/enhancer-binding protein alpha
TET2: the TET methylcytosine dioxygenase 2
HIP1: Huntingtin interacting protein 1
BAALC: the brain and acute leukemia cytoplasmic
ERG: ETS-related gene
ASXL1: additional sex combs like 1
